# Analysis of Caloric and Noncaloric Sweeteners Present in Dairy Products Aimed at the School Market and Their Possible Effects on Health

**DOI:** 10.3390/nu13092994

**Published:** 2021-08-27

**Authors:** Laura S. Briones-Avila, Mara A. Moranchel-Hernández, Daniela Moreno-Riolobos, Taísa S. Silva Pereira, Ana E. Ortega Regules, Karen Villaseñor López, Laura M. Islas Romero

**Affiliations:** Health Sciences Department, Universidad de las Américas Puebla, UDLAP, Ex-Hacienda Santa Catarina Mártir S/N, San Andrés Cholula 72810, Mexico; laura.brionesaa@udlap.mx (L.S.B.-A.); mara.moranchelhz@udlap.mx (M.A.M.-H.); daniela.morenors@udlap.mx (D.M.-R.); taisa.silva@udlap.mx (T.S.S.P.); ana.ortega@udlap.mx (A.E.O.R.); karen.villasenor@udlap.mx (K.V.L.)

**Keywords:** sweeteners, noncaloric sweeteners, food additives, sucrose, fructose, dairy products, children

## Abstract

Over the past decades, Mexico has become one of the main sweetener-consuming countries in the world. Large amounts of these sweeteners are in dairy products aimed at the children’s market in various presentations such as yogurt, flavored milk, flan, and cheeses. Although numerous studies have shown the impact of sweeteners in adults, the current evidence for children is insufficient and discordant to determine if these substances have any risk or benefit on their well-being. Therefore, this study aimed to describe the sweeteners present in 15 dairy products belonging to the school-age children’s market in Mexico and their impact on health. These dairy products were selected through a couple of surveys directed at parents of school-age children. After that, the list of ingredients of each product was analyzed to identify their sweetener content. From there, exhaustive bibliographic research on sweeteners and their possible health effects was carried out, which included 109 articles and 18 studies. The results showed that at a neurological, endocrinological, cardiovascular, metabolic, osseous, renal, hepatic, dental, reticular, carcinogenic, and gut microbiota level; sucrose, fructose, high-fructose corn syrup, maltodextrins, sucralose, and acesulfame K, have a negative effect. While maltodextrins, stevia, polydextrose, and modified starch have a positive one. For these reasons, it is necessary to evaluate the advantages and disadvantages that the consumption of each sweetener entails, as well as a determination of the appropriate acceptable daily intake (ADI).

## 1. Introduction

Sweeteners are all those substances or additives, other than monosaccharides, disaccharides, oligosaccharides, or sugars, that can give a characteristic sweet taste to the products of different industries, especially the food industry [1]. The foregoing allows generating a pleasant sensation on the consumer’s palate and arouse preference for these industrialized products, making them the most consumed from the first stages of life [1,2,3,4].

Regarding its classification, even it is not strictly regulated, in most cases, two factors are considered: the caloric content (caloric and noncaloric) and the origin of the sweetener (natural and artificial), as shown in Table 1 [5]. The sweeteners with the highest consumption in the industry are caloric sweeteners, such as sucrose, fructose, high-fructose corn syrup (HFCS), and polyols, which provide 4 kcal/g [6]. On the other hand, noncaloric sweeteners emerge as a strategy to reduce caloric sweetener intake and to prevent noncommunicable diseases (obesity, metabolic syndrome, and diabetes). Among this type of sweetener, sucralose and stevia stand out [2].

**Table 1:** Sweeteners’ classification (retrieved and modified from [7]).

**Caloric Sweeteners**		
Natural	Sugars	Sucrose, glucose, fructose, dextrose, lactose, maltose, galactose, trehalose, tagatose
	Caloric sweeteners	Honey, maple syrup, palm sugar, coconut sugar, sorghum syrup
Artificial	Modified sugars	High-fructose corn syrup, caramel, invert sugar
	Sugar alcohols	Sorbitol, xylitol *, mannitol, erythritol, maltitol, isomaltulose, lactitol, glycerol
**Noncaloric Sweeteners**		
Natural	Noncaloric	Stevia, thaumatin, pentadine, monelin, brazzein
Artificial	Noncaloric	Aspartame, sucralose, saccharin, acesulfame K, cyclamate, neotame

* Xylitol is also sometimes classified as a natural sweetener; however, it is classified here as an artificial sweetener because of how it is obtained for use as a sweetener.

Additionally, sweeteners have a property known as sweetening power. This quality is defined as “the grams (g) of sucrose that must be dissolved in water to obtain a liquid with the same taste as the solution of 1 g of sweetener in the same volume” [8] (p. 46).

The use of sweeteners in the industry dates back more than a century. The first sweetener was synthesized in 1879, representing the opportunity to create increased alternatives to sugar and different classes of sweeteners [9,10]. In the last three decades, there has been exponential growth in the production and demand for substitute sweeteners because of emerging globalization and marketing focused on light diet products. By 2010, the consumption of artificial caloric sweeteners such as high-fructose corn syrup and noncaloric artificial sweeteners such as aspartame reached values of more than 25 million tons [5].

In Mexico, the intake of sweeteners is around 9411 tons, placing it as one of the leading consuming countries in the world [5]. The noncaloric sweeteners with the highest ingestion in this country are saccharin, acesulfame K, stevia, aspartame, and sucralose [8,11]. On the other hand, concerning the most used caloric sweeteners, fructose, sucrose, and HFCS stand out [11].

Sweeteners’ quality and safety control are regulated internationally by institutions such as the Joint FAO/WHO Expert Committee on Food Additives (JECFA). At the national level, they are regulated by the Federal Commission for Protection against Sanitary Risks (COFEPRIS, by its acronym in Spanish) [12]. These institutions’ purpose is to determine the safe intake of sweeteners and other food by establishing the acceptable daily intake (ADI). However, even with these strict regulations and safety tests on caloric and noncaloric sweeteners, many concerns been raised about sweeteners’ adverse effects on consumers’ health, especially in children [12]. Regarding caloric sweeteners, many scientific studies relate the high intake of fructose, glucose, sucrose, honey, and HFCS with diabetes, obesity, metabolic syndrome, hepatic steatosis, nonalcoholic fatty liver, cardiovascular diseases, dental caries, and alterations in the intestinal microbiota [13,14,15,16]. On the other side, regarding noncaloric sweeteners, it has been found that sucralose is related to migraines and insensitivity to insulin, while aspartame is associated with prostate and breast cancer [17,18]. Nevertheless, it is necessary to highlight that research in children on short- and long-term effects has yielded contradictory results [10,15,19].

Unfortunately, in Mexico, large amounts of these sweeteners are in dairy products aimed at the children’s market in various presentations such as yogurt, flavored milk, flan, and cheeses. According to the 2018 National Health and Nutrition Survey (ENSANUT, by its acronym in Spanish) in Mexico, 38.2% of schoolchildren consumed sweetened dairy products [20]. Similarly, it has been shown that school-age children have a dietary inclination towards dairy products and that this consumption is related to overweight status [21].

It has been stated that the current evidence is insufficient and too discordant to determine if sweeteners present in dairy products have effects on Mexican school-age children’s health. Hence, this study aimed to describe the sweeteners present in dairy products on the children’s market in Mexico and their impact on health through a literary review to inform the risks and benefits of caloric and noncaloric sweetener consumption in school-age toddlers.

## 2. Materials and Methods

Thirty-seven dairy products offered to children in most supermarkets in Mexico were selected. The products were chosen from September to October 2020. Criteria considered for the selection of the products were their presentation, their publicity, the inclusion of cartoons, and eye-catching colors in the packaging and marketing. Then, a quick survey was applied to 36 parents with school-age children (6–12 years old) through digital sources such as social networks and social media. The survey was made through the Google Forms^®^ platform and included the 37 dairy products previously elected. Based on the survey results, the 15 most consumed dairy products were selected.

The first survey (applied to parents with school-age children) aimed to determine the dairy products (high in calories and sweetened) that are most consumed by children. This allowed us to delimit the products and design a second survey, which was applied to a broader group of parents, and through it relate the consumption of sweetened dairy products with nutritional health factors. The second survey was conducted through Microsoft Forms^®^. It included 41 questions (39 closed and 2 open); all questions were related to children’s nutritional status, eating habits, and ingestion of the 15 dairy products selected by the first survey. This last survey was applied from October 2020 to March 2021 and was sent out to 311 parents. It was completed by 225 parents who had school-age children; the remaining 86 parents either did not respond to the survey or had no children or children out of the school-age range. Similarly to the previous survey, the parents who participated were recruited from social network and social media, but the second survey was also distributed in an elementary school to parents who wanted to participate. It is necessary to clarify that the Ethics Committee of the affiliated institution approved the project. Moreover, in order to complete the surveys, participants had to accept a consent form in advance. Personal data (name, address) was not requested from any participant of the project.

Subsequently, we analyzed the list of ingredients and nutritional information of each of the 15 dairy products to identify their sweetener content. From there, we did exhaustive bibliographic research on sweeteners and their possible health effects.

The bibliographic analysis was conducted by three academics from November 2020 to March 2021 using the following databases: Ingenta connect (2004–Present), Redalyc (2002–Present), SciELo (1997–Present), PubMed (1996–Present), Springer Link (1842–Present), Elsevier (1880–Present), Google Scholar (2004–Present), ScienceDirect (1997–Present), ResearchGate (2008–Present), EBSCOhost (1984–Present), and Google Search (1997–Present).

We used search terms to explore all databases, where + represents AND and/represents OR: sweeteners/caloric sweeteners/noncaloric sweeteners/natural sweeteners/artificial sweeteners + health effect/children, ADI sweeteners, sucrose/fructose/high-dextrin/high-fructose corn syrup/sucralose/stevia/acesulfame k/polydextrose/modified starch + children/health effects. Regarding the search terms, “children” was used to achieve the purpose of this paper. However, not much information about the consumption of sweeteners by children, and not enough studies with children as participants, were found. For this reason, articles related to adult samples and animal samples were also included.

Selected books, scientific articles, and English and Spanish publications published predominantly after 2010 were inclusion criteria in order to provide an updated bibliographic review. We excluded those publications in other languages that lacked relevant information for this study, had a low grade of evidence, or both; this excluded studies with a high risk of bias or noncausal relationship, nonanalytical studies, or studies that included only unsubstantiated opinions. Articles were excluded after reading the title, abstract, or full text.

As for data extraction, the methodology followed by the authors was the critical reading and analysis of the chosen sources of information to subsequently synthesize the findings in a comprehensible way about the sweeteners found in dairy products and their respective positive and negative effects on the health of school-age children.

## 3. Results

From the 15 dairy products analyzed, the following sweeteners were identified: sucrose, fructose, high-fructose corn syrup, maltodextrins, sucralose, stevia, acesulfame K, and polydextrose. Furthermore, modified starch was considered because it is a nondigestible carbohydrate.

The analysis of the labeling of these 15 dairy products allowed us to identify the sweeteners contained in each of them, as shown in Table 2. In addition, the second survey elucidated the ten most consumed dairy products; these products are written in bold type in the first column of Table 2.

**Figure 1:**
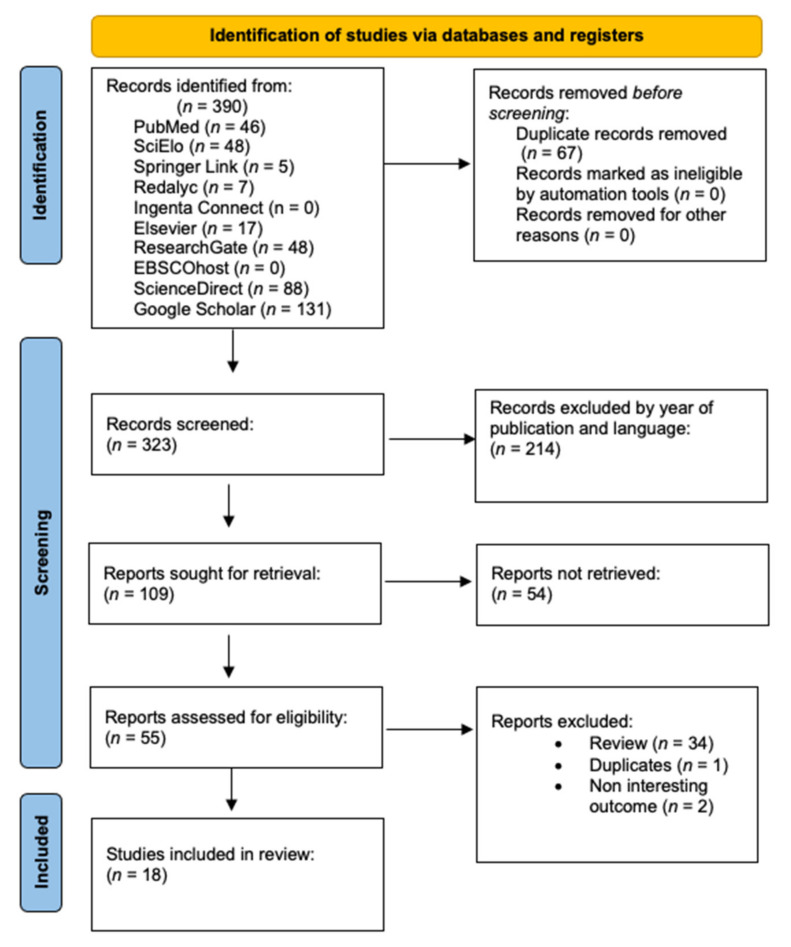
Prism diagram.

For the systematic review of this study, 390 articles were identified, from which 323 were selected for screening. After screening, 214 articles were removed because they did not comply with the inclusion criteria, such as year of publication (articles before 2010 that did not contain fundamental information) or publications in languages other than English or Spanish. Then, we eliminated 54 articles that lacked relevant information for this review, all based on the previously established inclusion criteria, as shown in Figure 1. The search effectuated by the three academics contained similarities since, before the investigation, a consensus was made of the databases that would be used and the search terms—resulting in a total of 18 studies published in Spanish and English languages.

Each of the sweeteners identified in the 15 dairy products analyzed has different characteristics (Table 3) and applications in the food industry that are broken down below.

**Table 3:** Main characteristics of caloric and noncaloric sweeteners [12,18,22,23,24,25,26,27,28,29,30,31,32,33,34,35,36].

Sweetener	Structure	Nomenclature	Sweetening Power	ADI, mg/kg/day
Sucrose	C_12_H_22_O_11_	Sucrose	1.0 (reference value)	“not specified”
Fructose	C_6_H_12_O_6_	Fructose	1.5–2.0	“not specified”
High-Fructose Corn Syrup	42–55% C6H12O6 and the rest C12H22O11	High-Fructose Corn Syrup or HFCS	42–110	“not specified”
Maltodextrins	C12H22O11	E-1400	-	“not specified”
Polydextrose	C12H22O11	E-1200	-	“not specified”
Sucralose	C12H19Cl3O8	E-955	600	5
Stevia	Variable	E-960	300	4
Acesulfame K	C4H4KNO4S	E-950	200	15

Acceptable daily intake (ADI).

### 3.1. Caloric Sweeteners Characteristics and Applications

#### 3.1.1. Sucrose

Sucrose is a disaccharide composed of equimolar amounts of glucose and fructose. This caloric sweetener is mainly extracted from sugar cane or beets, and it is used in the food industry as a sweetener, blocker of unpleasant flavors, preservative, energy source, and bulking agent. In addition, its sweetening power is used as a frame of reference to establish the degree of sweetness of other substances [15,37,38]. However, its ADI is determined as “not specified” by the JECFA [39].

#### 3.1.2. Fructose

Fructose or fruit sugar is a monosaccharide, the use of which has grown exponentially in the last 50 years, especially in carbonated beverages, because of its ability to decrease insulin response and act as a food stabilizer as well as its sweetening power of 1.5. In contrast with sucrose, it is a cheaper and more efficient sweetener due to the fact that a higher degree of sweetness is achieved with less product [13,15,18,40]. Additionally, the metabolization of this sweetener occurs in the liver, where it is subsequently used as a substrate in two metabolic pathways: glycolysis and gluconeogenesis [41].

#### 3.1.3. High-Fructose Corn Syrup (HFCS)

High-fructose corn syrup is a substance produced from cornstarch, and it has a composition of 42–55% fructose, with the rest being glucose. Its metabolization and excretion are equal to those of fructose [24]. Broadly speaking, it is a colorless, low-viscosity liquid with high sweetening power. It should be noted that different formulations depend on the amount of fructose they contain. In its presentation, HFCS 42 displays characteristics such as easy fermentation, freezing point control, and reduced crystallization; it is used to manufacture cookies, cereals, cereal bars, dressings, and sweets. HFCS 55, on the other hand, is used in concentrated juices, flavored waters, and carbonated beverages. Both types of HFCS are used to manufacture alcoholic beverages, yogurts, jams, and fruit in syrup [42]. HFCS 55 has a sweetening power of 100–110, and HFCS 42, 100 [32].

#### 3.1.4. Maltodextrins

Maltodextrins are made by acid or enzymatic hydrolysis of starches. These sweeteners in the food industry are frequently used to perform encapsulation, which protects against heat and humidity, giving stability, viability, nutritional value, and better appearance. Its use allows cost reduction and high effectiveness. Furthermore, these have low viscosity, are odorless and colorless, and do not affect the taste of the food in which is added [43].

#### 3.1.5. Polydextrose

Polydextrose is a polysaccharide synthesized by the polymerization of glucose. This sweetener is used in a wide range of baked products, confectionery, and beverages, since it provides texture and volume to food and has a low caloric value (1 kcal/g) and prebiotic properties [44,45]. Currently, the limit of consumption of polydextrose is not specified [46].

### 3.2. Noncaloric Sweeteners Characteristics and Applications

#### 3.2.1. Sucralose

Sucralose is a compound (1,6 dichloro-1,6 dideoxy-β-D-fructofuranosyl-4-chloro-4 deoxy-αD-galactopyranoside) obtained by the selective halogenation of the regular table sugar molecule (sucrose). In the gastrointestinal tract, only between 11 and 27% of sucralose is absorbed; the rest remains intact until excretion in the feces [35,47]. Sucralose’s ADI has been established as 5 mg/kg/day, and its sweetening power is considerably high at 600 [12,34]. Because of the sweetening potential of sucralose, and other properties such as its stability at high temperatures and its resistance to low pH, this sweetener plays an essential role in the structure, texture, and flavor of foods [15,48].

#### 3.2.2. Stevia

Steviol or stevia glycosides are a high purity extract from a native South American plant belonging to the Ateracea family, which is metabolized in the liver and excreted in the urine of humans [28,34]. This extract is used in the food industry, mainly in nonalcoholic beverages and baked goods [35]. Hence, it is 300 times sweeter than sucrose, heat stable, resistant to acid hydrolysis, nonfermentable, and odorless. Additionally, it has an ADI of 4 mg/kg/day [12,34,35].

#### 3.2.3. Acesulfame K

Acesulfame K is a sweetener with almost zero caloric value and a sweetening power 200 times greater than sucrose [12,34]. In the food industry, it is used in chewing gum, jellies, and baking products because it is soluble in water, maintains stability at high temperatures, and intensifies the sweet taste of food, presenting synergy with other sweeteners [3,12,35]. Even through it is not metabolized, it is absorbed in the small intestine and excreted through the urine. Therefore, the ADI is 15 mg/kg/day [12].

### 3.3. Modified Starch Characteristics and Applications

Because of its sheer size, whitish color, and mild flavor, modified starch is used in the industry as a thickener, stabilizer, and gelling agent. In dairy products such as yogurt, substituting 3% milk solids for starch suspensions increases viscosity and reduces syneresis [49]. Its ADI is listed as “not specified” by JECFA [50].

The following data present the health effects of the analyzed dairy products related to the aforementioned sweeteners and modified starch.

### 3.4. Caloric Sweeteners’ Health Effects

#### 3.4.1. Sucrose

Scientific evidence has reported adverse repercussions of sucrose (also known as table sugar) consumption on human health. Studies have shown that the sweetener promotes weight gain, cardiovascular diseases, type 2 diabetes mellitus, alterations in the hepatic metabolism of lipids, nonalcoholic fatty liver, changes in the intestinal microbiota, metabolic syndrome, and some types of cancer [51,52,53]. In a study carried out by Li et al. [52], it was described that the administration of a diet high in sucrose originated various physiological modifications in mice C57BL/6J. In the short term, the animals presented numerous metabolic affections, including obesity, hyperleptinemia, glucose intolerance, bone deterioration, peripheral insulin resistance, fasting hyperglycemia, and ectopic lipid deposition. Meanwhile, during prolonged exposure, the subjects exhibited morbid obesity, deposition of ectopic triglycerides in the liver and muscle, extensive loss of bone mass, sarcopenia, hyperinsulinemia, and impairment in short-term memory.

#### 3.4.2. Fructose

The scientific community has linked fructose intake with the presence of metabolic syndrome, diabetes, hypertension, vascular diseases, kidney lesions, glomerulosclerosis, proteinuria, and intestinal tubular fibrosis [54,55]. In addition, other studies have shown that the consumption of a diet high in fructose increases uric acid levels, leading to metabolic alterations such as dyslipidemia, insulin resistance, inflammatory arthritis, and gout. On top of that, every rise in ml/dl of uric acid increases the risk of developing diabetes by 17%. Similarly, this high uric acid concentration triggers endothelial dysfunction and excessive production of reactive oxygen species (ROS) [54,55,56]. On the other hand, it has been noted that the conversion of fructose to triose phosphate promotes gluconeogenesis, increases lipogenesis, and reduces beta-oxidation. As a result, a hyperglycemic state emerges and triglycerides and adiposity are elevated, which in turn triggers insulin resistance and obesity [57]. In a similar manner, an article published in 2015 stated that the consumption of this sweetener boosts the activity of various enzymes, the expression of proto-oncogenes, and inflammation, leading to cancer development [58].

#### 3.4.3. High-Fructose Corn Syrup

Recent studies have suggested that the negative metabolic effects of high fructose consumption also occur with HFCS intake. For example, a study applied to healthy young humans showed that consuming 25% of their total energy intake from HFCS for two weeks increased fasting LDL and apoB levels, as well as postprandial triglycerides. On the other hand, in a more comprehensive study, it was observed that a 20% rise of HFCS in the energy requirements of overweight and obese subjects caused an increment in liver lipids, cholesterol, and muscle fat [59]. Furthermore, a study carried out by Patterson et al. [24] showed an extension in serum glucose after HFCS consumption in lean, overweight, or obese subjects (without diabetes) and individuals with type 2 diabetes mellitus without complications. Although other research has supported these results, it has been previously clarified that fructose and sucrose consumption significantly increase glycosidic levels and decrease or maintain insulin parameters. In addition, some researchers have speculated that the intake of HFCS may contribute to kidney and cardiovascular diseases in humans [55,59].

#### 3.4.4. Maltodextrins

The intake of maltodextrins has reported beneficial and harmful effects in mammals. For example, in a study carried out in 2020, the application of standardized care and the implementation of carbohydrate loads were compared on the duration and severity of postoperative hyperglycemia in diabetic and nondiabetic patients undergoing cardiac surgeries. The results showed that a carbohydrate load combined with maltodextrin and citrulline significantly reduced the rates of hyperglycemia after cardiac surgery [60]. Similarly, in a double-blind clinical study applied to 150 obese patients, the effect of maltodextrin as a prebiotic on the clinical outcome of these subjects was investigated. It was observed that the administration of this ingredient helped reduce body mass index (BMI), caloric intake, systolic and diastolic pressure, insulinemia, and AST [61]. In contrast, various experiments in mice have indicated that excessive intake of maltodextrins can lead to faster weight gain, larger retroperitoneal fat pads, and poor spatial cognition [62]. Likewise, the results of an investigation in which 5% concentrated maltodextrose was given to mice for 45 days showed that a diet high in maltodextrins reduces the intestinal content of the mucin-2 secreting glycoprotein and promotes the adherence of pathogenic bacteria to the intestinal epithelium, which may contribute to intestinal disease [63].

#### 3.4.5. Polydextrose

Scientific evidence regarding polydextrose has shown only beneficial consequences on human health, since it helps to modulate appetite and satiety, causing a reduction in total caloric input [64,65,66]. Furthermore, when this sweetener is added to food, apart from improving textural properties, it acts as a prebiotic and increases antioxidant, antihypertensive, and antidiabetic activity [67,68].

### 3.5. Noncaloric Sweeteners’ Health Effects

Some caloric sweeteners indeed contribute to human well-being. However, the large number of unfavorable effects reported by these substances has caused the need to reduce their consumption in a generalized way, giving room for the noncaloric sweeteners to emerge [69]. Since the discovery of noncaloric sweeteners, their industrial use, production, and diversity have increased. Today they can be found in 6000 food products around the world [12,70].

#### 3.5.1. Effects of Sweeteners towards Health in Living Beings

So far, it has not been clarified if there is a relationship between the consumption of noncaloric sweeteners and an increase in the development of diseases in children. Although numerous studies have shown that these sweeteners are a better option than caloric ones, this does not imply that noncaloric sweeteners are exempt from harm to human health [10].

Various studies and literary evidence have found multiple effects, both positive and negative, of noncaloric sweeteners in living beings, which are mentioned below.

##### 3.5.1.1. Gut Microbiota

Sucralose consumption decreases anaerobic and aerobic bacteria, such as Bifidobacterium, Bacteroides, Lactobacillus, and Clostridium, triggering dysbiosis. In more recent studies, it was stated that sucralose in mice modified the microbiota in 14 different taxonomic levels, among which were Lachnospiraceae, Ruminococcaceae, and Verrucomicrobiaceae. In addition, changes were observed in the synthesis and regulation of amino acids [28]. As for acesulfame K, research carried out in 2017 indicated few effects on mouse intestinal microbiota and metabolism. In contrast, another study carried out by Bian et al. [71] declared that the ingestion of acesulfame K for four weeks altered the intestinal microbiota in mice. These contradictory outcomes are due to the amount of acesulfame K administered; in the first study, the dose was 15 mg/kg (maximum ADI level), while in the second, it was 37.5 mg/kg (twice the limit of the ADI) [28].

It has been documented that the roots of S. Rebaudiana, whence the stevioids are extracted, contain functional ingredients that generate a positive effect on human health. One of these compounds, fructans, improves the growth of some microbial strains that play an essential role in gastrointestinal function [28]. However, the number of studies aimed at elucidating the influence of noncaloric sweeteners on the intestinal microbiota is minimal. So far, no specific area has been developed to study their impact on children [10].

##### 3.5.1.2. Glucose Metabolism

There have been multiple studies in humans, animals, and in vivo that showed that stevia decreases serum levels of glucose, serum triglycerides, urea nitrogen, serum creatinine, total cholesterol, HDL, and LDL in diabetic rats and mice. In a human clinical trial, stevia was involved in glucose modulation by lowering postprandial glucose and insulin levels. Other research has demonstrated that this natural sweetener increases insulin sensitivity, reduces gluconeogenesis, and prevents oxidative DNA damage related to type 2 diabetes mellitus [72]. In contrast, acesulfame K has been associated with a 20–30% growth in glucose intake, which may worsen postprandial hyperglycemia in individuals with type 2 diabetes mellitus [73]. Conversely, scientific evidence has shown that long-term intake of sucralose does not generate adverse effects on glucose homeostasis or insulin responses [74].

##### 3.5.1.3. Cancer

In respect to the relationship between the noncaloric sweeteners studied and the risk of cancer, certain types act beneficially and others harmfully.

Stevia and its polyphenols have anticancer, antioxidant, anti-inflammatory, antidiabetic, antihypertensive, antimicrobial, and antilipid properties [75,76].

On the contrary, doubts have arisen regarding the genotoxicity of acesulfame K. On the one hand, most biological tests have shown that it lacks carcinogenicity in humans, and although studies have shown that a commercial combination between acesulfame K and aspartame produces genotoxic activity, only aspartame is responsible for this toxicity [77,78]. On the other side, however, acesulfame K, when broken, generates acetoacetamide, which is toxic to the human body [79].

Regarding sucralose, though it is considered harmless and without the genotoxic potential to produce genetic damage or cancer in humans, a study mentioned that because of the conditions that this sweetener causes on the intestine (the inactivation of digestive proteases, the microbiota, and the intestinal barrier), it can promote the development of colorectal cancer [80].

##### 3.5.1.4. Dental Health

Stevia has been documented to possess anticariogenic and antiperiodontopic properties that position it as an ideal substitute for sucrose consumption. In addition, in vivo and in vitro studies have postulated that it has antibacterial activity on microorganisms that favor the presence of dental caries and a low acidic potential within the dental film that prevents the appearance and progression of cavities [81].

##### 3.5.1.5. Fat Tissue and Weight Gain

Although acesulfame K stimulates adipogenesis and inhibits lipolysis in cells 3T3-L1 [10], in a clinical trial performed in children aged 4 to 11 years, it was observed that there was less weight gain in the group that consumed a drink with sucralose and acesulfame K than in the group that ingested a sugary drink [82]. In contrast, the effect of stevia on adipose tissue has not been thoroughly studied [10].

##### 3.5.1.6. Nervous System

Several in vitro and in vivo studies have suggested that acesulfame K has the potential to decrease intracellular ATP production and reduce the protective activity of neuronal cells. Furthermore, other research in mice showed that chronic use of acesulfame K has a negative impact on memory and learning. However, these studies were carried out with doses higher than those established by legislation [73]. Meanwhile, sucralose consumption has been linked to increased food ingestion by indirect and direct stimulation of taste receptors [83]. Likewise, in a study carried out by Farid et al. [83], sucralose and stevia were shown to pose different risks to consumers. The former caused glucose and liver enzyme levels to increase, while the latter raised creatinine and urea levels, decreased antiinflammatory cytokines, and heightened the number of proinflammatory cytokines.

### 3.6. Modified Starch

It has been found that modified starch, during its colonic fermentation by the intestinal microbiota, produces short-chain fatty acids such as acetate, propionate, and butyrate that favor intestinal health by increasing the function of the intestinal barrier and reducing inflammation and the risk of colon cancer. Furthermore, these fatty acids decrease the release of glucose into the blood; reduce the incidence of lipid and leptin resistance, serum cholesterol, and LDL levels; and elevate the YY peptide, which plays a vital role in appetite—making modified starch a good option for patients with obesity or diabetes. On top of that, in patients with chronic kidney disease, modified starch favorably modulates the intestinal microbiota and reduces toxic biomarkers and inflammation [84].

More data and information collected on the health effects of the sweeteners mentioned and of modified starch are summarized in the following table (Table 4).

## 4. Discussion

### 4.1. Epidemiology of Overweight and Obesity in Mexico

In the last four decades, there has been an increase in the prevalence of obesity in Mexico. This evolution is evidenced by the ENSANUT every six years (2000, 2006, 2012, 2018) through the implementation of questionnaires at the national level. In its latest report, carried out in 2018, it avouched that the percentage of overweight and obesity in adults was heightened by 3.9% compared to the previous six years, at a total of 75.2%. Similarly, the data showed an increment in the prevalence of overweightness and obesity in children between 5 and 11 years due to the worsened of individual obesity rates (+2.9%) [20,100]. Overweightness and obesity represent a risk factor for developing chronic noncommunicable diseases, such as diabetes mellitus, arterial hypertension, dyslipidemia, and various types of cancer, and have negative impacts on morbidity, mortality, and the economy of the Mexican population [101].

The growth in overweight, obesity, and chronic noncommunicable disease scales has been prompted by the transformation in the Mexican diet from fresh and unprocessed foods to ultraprocessed foods high in sodium, sugar, and fat aliments. Currently, an enormous amount of the total energy consumed by a Mexican comes from ultraprocessed foods (23.1%). Mexico is also one of the countries with the highest consumption of sugar-sweetened beverages; in fact, such beverages represent 70% of added sugar intake in the diet [100]. According to official figures, Mexico ranks sixth in sugar consumption worldwide, summing around 7832 million tons per year [102].

### 4.2. Knowledge of Noncaloric Sweeteners

It is important to note that, although these noncaloric sweeteners are widely positioned in the food industry, knowledge about their physicochemical characteristics and metabolic effects is scarce among both the general population and health professionals. In 2016, a semistructured survey was applied to 1183 health specialists who attended the XXXIX National Congress of Internal Medicine (in Mexico) to evaluate their degree of expertise regarding noncaloric sweeteners, their biological and physicochemical characteristics, metabolic effects, uses, and recommendation rates. The results obtained displayed that only 69% of respondents could cite the correct definition of noncaloric sweeteners, 30% identified them as artificial compounds, 3% knew that each noncaloric sweetener has a specific chemical structure, 4% understood their uses for nondiabetic patients, 2% knew that they could be used for weight loss, and 51% believed that they alter glucose homeostasis. Despite these outcomes, there was a more significant trend towards the recommendation of noncaloric sweeteners (74%) by these professionals. The preceding shows that a large proportion of health specialists do not have enough information regarding these compounds. Therefore, it is essential to implement educational programs on the subject to provide up-to-date and scientifically supported information to avoid promoting behaviors that threaten the health of individuals [103].

In comparison, the Salvador Zubirán National Institute of Medical Sciences and Nutrition (INCMNSZ) carried out a cross-sectional study involving 150 patients with diabetes. The results showed that 62.7% knew what noncaloric sweeteners were and 44% considered that the information they had on the subject was insufficient; furthermore, of those who consumed these sweeteners, 51.3% said health professionals had recommended them and 53.3% received the suggestion by a family member or friend [104]. In 2016, the ENSANUT carried out a cross-sectional, probabilistic, and multistage study on the general Mexican population. The statistical data reported that only 40.6% of the interviewees read the nutritional labels of industrialized products. The nutritional table was the most read (71.6%), followed by the daily food guides (known as the Guideline Daily Amount in English) (55.9%) and last by the list of ingredients (26%) [105], where information on added sweeteners is found.

### 4.3. Acceptable Daily Intakes in Children

Over this time, concerns have also arisen in connection with food safety and its implications on human well-being. The American Diabetes Association (ADA) states that the use of noncaloric sweeteners is safe when they are consumed in the amounts pre-established by the Food and Drug Administration (FDA) and JECFA, referring to the ADI. However, different studies carried out in Europe and Latin America estimated that the consumption of these sweeteners presents an increased prevalence and a risk of exceeding the ADI in children and adolescents, mainly because the ADI is predicted as consumed milligrams of a sweetener according to an individual’s kilograms of weight, and a child’s body weight is less than that of an adult [69,104]. A descriptive and cross-sectional study performed in Argentina in 2011 exhibited that 51% of the children and adolescents studied (2664) consumed foods or beverages that contained some noncaloric sweetener; of these beverages, 95% provided aspartame, 90% acesulfame K, 69% cyclamate, 66% saccharin, and 72% sucralose.

Conversely, 53% of Argentine school-age children consumed some type of sweetener, exceeding the acceptable daily intake of cyclamate by 0.1%. Similarly, some research executed in Denmark, Sweden, and Italy disclosed that the toddler population exceeded the ADI of saccharin and cyclamate. An evaluation carried out on Chilean children from 6 to 14 years old exhibited that 100% of the children studied consumed at least one noncaloric sweetener: most frequently sucralose (99.6%), followed by acesulfame K (95.6%), aspartame (92.4%), cyclamate (29.2%) and saccharin (11.2%). However, the ADIs were not exceeded [106]. In Mexico, the ingestion of high-fructose corn syrup and sugar has been estimated. However, the intake of noncaloric sweeteners has not been studied [104,107], nor has their impact on school-age children.

### 4.4. Ingredients, Advertising, and Strategies Regarding Dairy Product

In recent years, various dairy products have appeared aimed at Mexican children that, as part of their ingredients, contain modified starch and sweeteners (caloric and noncaloric). The market promotes these items to children through advertisements and eye-catching packaging. Different systematic reviews over the years have determined the direct impact of advertising on nutritional knowledge, preferences, shopping behaviors, and the eating pattern of toddlers; advertising normalizes harmful eating habits and increases the demand for unhealthy products, which leads to elevated rates of overweight and obesity [108,109]. Despite this, it is essential to highlight the creation of different public health strategies that seek to improve the current situation regarding the consumption of these products. For example, there is the most recent change to the Official Mexican Standard NOM-051-SCFI/SSA1-2010, which, through labeling alerts, makes the consumer aware that a product containing sweeteners is not recommended for children, since an early introduction of these sweetener additives to the diet could increase the probability of presenting the effects described previously.

### 4.5. Authors’ Observations

Essentially, we have to take in account that all the caloric and noncaloric sweeteners reviewed in the study, if consumed within the recommended intake limits and if the consumer’s health and nutrition status are optimal, are considered safe. However, the authors recommend taking special precautions with sucrose, fructose, high-fructose corn syrup, maltodextrins, sucralose, and acesulfame K due to their documented negative effects on well-being.

On the other hand, the review reported in this writing combined data from studies, publications, surveys, reviews, and meta-analyses to identify the sweeteners present in dairy products destined for the Mexican children’s market and their impact on well-being. This systematic review has some limitations, such as lingual restriction. In the search for sources of information (limited only to publications in Spanish and English), studies were scarce regarding the long- and short-term health effects of caloric and noncaloric sweeteners in children; the quality of these studies and the data they contained varied; and information was limited on the consumption, advertising, and beliefs of caloric and noncaloric sweeteners in Mexico. Because of this limitation, it is necessary to state that although the principal interest of this review was the evaluation of the possible health effects of sweeteners present in dairy products targeted at children, most of the evidence sourced from human studies was found by studying adults. For this reason, it is necessary to highlight that knowledge about the effects of sweetener consumption in childhood is deficient and requires more research (particularly on how sweetener consumption early in life is related to health conditions and eating habits in adulthood).

In addition, it is necessary to mention that in the animal studies mentioned throughout the text, the animals used were rats and mice. Some female or pregnant rats/mice were used, but most of the rats/mice of the population studied were male. Furthermore, in each of the animal studies, the age of the animals was described, but in general, the animals used were 3–16 weeks old when the studies were in the initial phase.

However, this review also contains numerous strengths. The first is presenting novel information about the sweeteners contained in dairy products offered to the children’s sector in Mexico. In addition, knowledge about sweeteners that is possessed in the professional or general fields generates social relevance concerning the consumption of these products in the Mexican population and consequently a practical implication in different fields of health and training of better eating habits by consumers. Finally, we can affirm that one of the most important contributions of this article is encouraging future lines of research in five areas: (1) The effect of sweeteners in children in the short and long term. (2) The impact on the health of Mexican children by sweetened dairy products. (3) The exceeding, or not, of the ADI of different sweeteners in the Mexican child population. (4) Elucidation of parents’ knowledge about the impact of dairy items. (5) The impact of Mexican marketing on the choice of dairy products.

## 5. Conclusions

So far, it can be said that both caloric and noncaloric sweeteners present in dairy products aimed at the children’s market in Mexico can contribute to the development of various diseases (overweight, obesity, morbid obesity, dyslipidemia, metabolic syndrome, diabetes, arterial hypertension, hepatic steatosis, nonalcoholic fatty liver, hyperleptinemia, sarcopenia, inflammatory arthritis, and some types of cancer). Moreover, they can generate side effects that can be considered harmful (dental caries, migraines, alterations to the intestinal microbiota and metabolism, bone deterioration, and impairment in short-term memory). However, other sweeteners can favor the state of health. For example, adequate intake of maltodextrins favors prebiotic properties and lowers blood sugar levels and blood pressure. Similarly, polydextrose has antioxidant, antihypertensive, antidiabetic, and prebiotic properties and helps to regulate satiety. Meanwhile, stevia has been shown to have a glucose-regulating, antioxidant, anticancer, antibacterial, antihypertensive, antiperiodontic role as well as helping to control satiety. Also, it is known that sucralose tends to reduce weight gain and does not alter glycemic levels. Modified starch promotes intestinal health, reduces blood glucose, and favorably modulates the lipid profile. For this reason, it is necessary to evaluate the advantages and disadvantages that the consumption of each sweetener entails, in addition to determining whether the ADI currently regulated by the FDA is safe and, if not, estimating the actual intake values in children, to avoid the development of the aforementioned adverse effects.

To address the uncertainty generated by the ingestion of sweeteners in dairy products (aimed at the school-age market), it is essential to promote policies that regulate their use and consumption in the food industry. In this way, the employment of sweeteners with favorable health effects can be increased. On the other hand, school-age children must have adequate nutritional education at all levels of primary education so they can be informed to choose better products and generate better eating habits throughout their lives.

Finally, the data obtained from the conducted surveys will be analyzed and used in other articles related to children’s nutritional status in Mexico.

## Figures and Tables

**Table 2:** Sweeteners contained in the dairy products selected for the study.

Dairy Product Description	Contained Sweeteners		Modified Starch
	Caloric	Noncaloric	
**Petit Suisse cheese**	Sucrose, fructose	-	-
**Fermented dairy product with fruit (medium presentation)**	Sucrose, maltodextrin	-	Yes
**Dairy product ferment fruit flavor (small presentation)**	Sucrose, maltodextrin	-	Yes
**Fruit flavored yogurt with cereal**	Sucrose, maltodextrin, high-fructose corn syrup	Sucralose	Yes
**Fermented dairy product with *Lactobacillus casei* Shirota**	Sucrose	-	-
**Ultrapasteurized reconstituted skim milk with flavorings**	Sucrose, maltodextrin, polydextrose	Stevia	-
**Ultrapasteurized semiskimmed milk with ice cream flavor (boxed presentation)**	Sucrose	-	-
Ultrapasteurized semiskimmed milk with ice cream flavor (bottle presentation)	Sucrose	-	-
**Chocolate milk**	Sucrose	Sucralose, acesulfame K, stevia	-
Yogurt with fruit preparation	Sucrose, high-fructose corn syrup	Acesulfame K, sucralose	Yes
Sweetened natural yogurt with cereal	Sucrose	-	Yes
Plain whipped yogurt	Sucrose, Fructose	Acesulfame K	Yes
**Custard (*Dulce de Leche*)**	Sucrose	-	Yes
Flan	Sucrose, fructose	-	Yes
**Petit Suisse cheese (designed to be eaten by smashing it)**	Sucrose, fructose, maltodextrin	-	Yes

The ten most consumed dairy products, elucidated in the second survey, are written in bold.

**Table 4:** Publications related to sweeteners and their health effects.

Sweetener	Study Reference	Title of Publication	Country	Subjects	Exposure	Conclusions
Sucrose	[85]	“Impact of dietary sucrose on adiposity and glucose homeostasis in C57BL/6J mice depends on mode of ingestion: liquid or solid”	China	Male C57BL/6J mice	Sucrose in solid and liquid food (rodent chow and drinking water)	In this study, it was found that mice that consumed sucrose-sweetened liquids had more significant body weight gain and presented a reduction in the response of insulin. Thus, the consumption of sucrose-sweetened liquids can induce the development of metabolic syndrome and changes in body weight.
	[86]	“High Sucrose Intake at 3 Years of Age Is Associated with Increased Salivary Counts of Mutans Streptococci and Lactobacilli, and with Increased Caries Rate from 3 to 16 Years of Age”	Finland	Children from 3 to 16 years old	Fructose added in diet or food	Children with high sucrose intake are more likely to develop caries and have higher salivary counts of mutans streptococci.
	[87]	“Dietary sugars and noncaloric sweeteners elicit different homeostatic and hedonic responses in the brain”	Netherlands	16 healthy men from 18 to 25 years of age with body mass index between 20–23 kg/m^2^	50 g glucose, fructose, or sucrose, or 0.33 g sucralose dissolved in 300 mL of water	This research studied the metabolic responses of sweeteners; it was found that sucrose, like glucose, can induce an elevation of glucose and insulin in blood after ingestion, and that sucrose had a delayed and lesser response from the hypothalamus.
	[88]	“A randomized controlled trial contrasting the effects of 4 low-calorie sweeteners and sucrose on body weight in adults with overweight or obesity”	United States of America	Individuals from West Lafayette	Beverages sweetened with 100, 120, or 140 g sucrose	The consumption of sucrose can induce gain of body weight in comparison to noncaloric sweeteners.
	[89]	“Sucrose-sweetened beverages increase fat storage in the liver, muscle, and visceral fat depot: a 6-mo randomized intervention study”	Denmark	Healthy, nondiabetic subjects between 20 and 50 years old with BMI 26–40 and blood pressure < 160/100 mm Hg.	Beverages sweetened with sucrose, aspartame, saccharin, sucralose, or rebaudioside A	The consumption of beverages sweetened with sucrose or fructose for six months can increase adipose tissue accumulation compared to other beverages such as noncaloric milk and soda. Also, the results of human studies suggested that the excess consumption of fructose produces adverse metabolic changes, such as gain of adipose tissue, increased triglycerides, decreased insulin sensitivity, and dyslipidemia.
Fructose	[90]	“Long-term fructose feeding changes the expression of leptin receptors and autophagy genes in the adipose tissue and liver of male rats: a possible link to elevated triglycerides”	Finland	Sprague-Dawley rats	Fructose-rich diet	This study demonstrated that fructose consumption can induce leptin resistance, elevate triglyceride levels, and induce insulin resistance and hepatic steatosis.
	[91]	“Fructose increases risk for kidney stones: potential role in metabolic syndrome and heat stress”	United States of America	Healthy male adults between 40–65 years of age	200 g fructose daily in water	The results of this study showed that fructose consumption appears to increase the formation of urinary stones by the direct effects on urinary pH, overproduction of oxalate, and the increment of serum levels of uric acid.
	[92]	“Efectos del consumo elevado de fructosa y sacarosa sobre parámetros metabólicos en ratas obesas y diabéticas”	Argentina	Overweight and diabetic IIMb/β rats	Diets with fructose, sucrose, or corn starch	Cholesterol and triglyceride levels were elevated in the groups fed with fructose and sucrose. In addition, mild hepatic steatosis and other severe effects such as obesity and overweight, insulin resistance, diabetes type 2, hyperuricemia, nonalcoholic fatty liver, and renal failure were demonstrated.
High-Fructose Corn Syrup	[93]	“A dose-response study of consuming high-fructose corn syrup–sweetened beverages on lipid/lipoprotein risk factors for cardiovascular disease in young adults”	United States	187 adults between 18 and 40 years of age and with BMI 18–35 kg/m^2^	Beverages sweetened with high-fructose corn syrup	The results of the study mentioned that the consumption of beverages sweetened with HFCS is associated with an increase of uric acid serum levels and dose-response increases of circulating triglycerides and lipoprotein risk factors for CVD.
	[94]	“High fructose corn syrup induces metabolic dysregulation and altered dopamine signaling in the absence of obesity”	United States	Male and female C57BL/6 m mice	High-fructose corn syrup in chow diet or a 10% solution of HFCS-55 in drinking water	The research claimed that changes in body weight were not found. However, the data showed that the consumption of high-fructose syrup can induce metabolic dysregulation associated with glucose serum levels and dopamine independent of obesity or body weight gain.
Maltodextrins	[62]	“Maltodextrin can produce similar metabolic and cognitive effects to those of sucrose in the rat”	Australia	Male albino Wistar rats	Sucrose, maltodextrin solution, or drinking water	In this study, the data suggested that the excess consumption of maltodextrin produces rapid body weight gain and adipose tissue accumulation, like the consumption of sucrose.
Polydextrose	[44]	“El consumo de la polidextrosa previene la obesidad y sus comorbilidades en ratas alimentados con dieta hypercaloric”	Brazil	Male Wistar Rats	Hypercaloric diet with polydextrose supplementation	In this study, no effects of polydextrose associated with cholesterol and lipoproteins were detected. However, it was mentioned that polydextrose can affect glucose metabolism by lowering insulin and glucose serum levels, improving the metabolic response.
Sucralose	[95]	“Short-term impact of sucralose consumption on the metabolic response and gut microbiome of healthy adults”	Chile	Healthy men between 18–50 years with a BMI between 20–30 kg/m^2^	Sucralose capsules (260 mg)	The study results showed that participants who consumed sucralose presented lesser body weight gain compared to the placebo group. Moreover, it was confirmed that sucralose consumption does not alter glycemic control, and there were no changes in the gut microbiome.
Stevia	[96]	“Effects of stevia, aspartame, and sucrose on food intake, satiety, and postprandial glucose and insulin levels”	United States	19 healthy individuals with BMI between 20 and 24.9 kg/m^2^ and 12 individuals with BMI between 30 and 39.9, all 18–50 years of age	Preload with stevia before lunch and dinner	In the study, the consumption of stevia had different effects in comparison to sucrose after the preload of stevia, insulin, and glucose serum levels were reduced. Also, participants who consumed stevia preload reported similar levels of satiety compared to when they consumed sucrose preload.
	[97]	“ Effects of Stevia Extract on Postprandial Glucose Response, Satiety and Energy Intake: A Three-Arm Crossover Trial”	United Kingdom	20 women and 10 men between 18 and 65 years of age with BMI between 18.5 and 29.9 kg/m^2^	Preload containing water mixed with 1 g stevia	After the ingestion of the preload, glucose serum levels were not as elevated as the for the preload containing sucrose and water. Also, it was proven that the consumption of stevia reduces appetite and keeps satiety.
Acesulfame K	[71]	“The artificial sweetener acesulfame potassium affects the gut microbiome and body weight gain in CD-1 mice”	United States	Male CD-1Mice	Noncaloric sweetener: acesulfame K	This study found that the consumption of acesulfame K in male CD-1 mice can increase body weight gain. Furthermore, both genders presented shifts in the gut bacterial community composition.
	[98]	Consumption of a Carbonated Beverage with High-Intensity Sweeteners Has No Effect on Insulin Sensitivity and Secretion in Nondiabetic Adults	France	Nondiabetic adults with BMI between 19 and 29 kg/m	Carbonated beverages containing 129 mg aspartame and 13 mg acesulfame K	The data showed that after 12 weeks of consuming carbonated beverages sweetened with acesulfame K, participants did not present insulin sensitivity or insulin secretion alterations.
Modified Corn Starch	[99]	“Endurance exercise in a rat model of metabolic syndrome”	Australia	Male Wistar rats	Diet rich in modified corn starch or HCHF diet	Rats fed with a diet high in carbohydrates and fat presented metabolic alterations such as obesity and dyslipidemia, impaired glucose tolerance, and increased systolic blood pressure compared to those fed with a diet based on modified corn starch.

## Data Availability

No new data were created or analyzed in this study. Data sharing is not applicable to this article.

## References

[1] Wakida-Kuzunoki G.H., Aguiñaga-Villaseñor R.G., Avilés-Cobián R., Baeza-Bacab M.A., Cavagnari B.M., Castillo-Ruíz V., Hernández-Aguilar J.C., López-García R., Martínez-Ramos A., Martínez-Rodríguez N. (2017). Edulcorantes no calóricos en la edad pediátrica: Análisis de la evidencia científica. Rev. Mex. Pediatr..

[2] Manzur-Jattin F., Morales-Nuñez M., Ordosgoitia-Morales J., Quiroz-Mendoza R., Ramos-Villegas Y., Corrales-Santander H. (2020). Impacto del uso de edulcorantes no calóricos en la salud cardiometabólica. Rev. Colomb. Cardiol..

[3] Calzada-León R., Ruíz-Reyes M.L., Altamirano-Bustamante N., Padrón-Martínez M.M. (2013). Uso de edulcorantes no calóricos en niños. Acta Pediatr. Mex..

[4] Gil-Campos M., San José González M.A., Díaz Martín J.J. (2015). Uso de azúcares y edulcorantes en la alimentación del niño. Recomendaciones del Comité de Nutrición de la Asociación Española de Pediatría. An. Pediatr..

[5] Santillán A., García L.A., Vásquez N., Santoyo V.H., Melgar M., Pereira W., Larrahondo J.E., Merino A. (2017). Impacto de la Sustitución del Azúcar de caña por Edulcorantes de Alta Intensidad en México.

[6] Alejos de Domingo A. (2018). Edulcorantes o Azúcar: Efectos Sobre la Salud.

[7] García-Almeida J.M., Casado G.M., García J. (2013). Una visión global y actual de los edulcorantes: Aspectos de regulación. Nutr. Hosp..

[8] Bulman J.F., Navarro J., Díaz E., Guzmán-Valdivia G., Rodriguez F. (2018). Ingesta de edulcorantes no nutritivos en tres poblaciones distintas de adultos en México. Rev. Chil. Nutr..

[9] Velasco J. (2019). Efectos de los Edulcorantes Artificiales Sobre la Salud.

[10] Durán S., Angarita L., Escobar M.C., Rojas D., de Assis J. (2018). Noncaloric Sweeteners in Children: A Controversial Theme. BioMed Res. Int..

[11] Gobierno de México https://www.gob.mx/cms/uploads/attachment/file/201026/Nota_Febrero.pdf.

[12] Aldrete-Velasco J., López-García R., Zúñiga-Guajardo S., Riobó-Serbán P., Serra-Majem L., Suverza-Fernández A., Esquivel-Flores M.G., Molina-Segui F., Pedroza-Islas R., Rascón-Hernández M. (2017). Análisis de la evidencia disponible para el consumo de edulcorantes no calóricos. Documento de expertos. Med. Int. Méx..

[13] Carvallo P., Carvallo E., Barbosa-da-Silva S., Mandarim-de-Lacerda C.A., Hernández A., del Sol M. (2019). Efectos metabólicos del consumo excesivo de fructosa añadida. Int. J. Morphol..

[14] Riveros M.J., Parada A., Pettinelli P. (2014). Consumo de fructosa y sus implicaciones para la salud: Malabsorción de fructosa e hígado graso no alcohólico. Nutr. Hosp..

[15] Romo A., de Haro F., Fuentes O. (2018). Edulcorantes energéticos y no energéticos: Utilidad y Efectos secundarios. Nutrición en Gastroenterología: Aspectos clínicos y dietéticos.

[16] Bejarano J.J., Suárez L.M. (2015). Algunos peligros químicos y nutricionales del consumo de los alimentos de venta en espacios públicos. Rev. Univ. Ind. Santander. Salud..

[17] Stephens-Camacho N.A., Valdez-Hurtado S., Lastra-Zavala G., Félix-Ibarra L.I. (2018). Consumo de edulcorantes no nutritivos: Efectos a nivel celular y metabólico. Perspect. Nut. Hum..

[18] Academia https://www.academia.edu/22561760/Glucosa_and_Fructosa.

[19] Lohner S., Toews I., Meerpohl J.J. (2017). Health outcomes of non-nutritive sweeteners: Analysis of the research landscape. Nutr. J..

[20] INEGI https://www.inegi.org.mx/contenidos/programas/ensanut/2018/doc/ensanut_2018_presentacion_resultados.pdf.

[21] Shamah-Levy T., Cuevas-Nasu L., Méndez-Gómez-Humarán I., Jimenez-Aguilar A., Mendoza-Ramírez A.J., Villalpando S. (2011). La obesidad en niños mexicanos en edad escolar se asocia con el consumo de alimentos fuera del hogar: Durante el trayecto de la casa a la escuela. Arch. Latinoam. Nutr..

[22] PubChem-National Library of Medicine—Sucrose. https://pubchem.ncbi.nlm.nih.gov/compound/Sucrose.

[23] PubChem-National Library of Medicine—D-Fructose. https://pubchem.ncbi.nlm.nih.gov/compound/2723872.

[24] Patterson M.E., Yee J.K., Wahjudi P., Mao C.S., Lee W.P. (2018). Acute metabolic responses to high fructose corn syrup ingestion in adolescents with overweight/obesity and diabetes. J. Nutr. Intermed. Metab..

[25] PubChem-National Library of Medicine—Maltodextrin-dextrose equivalent 10-15. https://pubchem.ncbi.nlm.nih.gov/compound/68229136.

[26] PubChem-National Library of Medicine—Melibiose. https://pubchem.ncbi.nlm.nih.gov/compound/872.

[27] PubChem-National Library of Medicine—Sucralose. https://pubchem.ncbi.nlm.nih.gov/compound/71485.

[28] Ruiz F.J., Plaza J., Saéz M.J., Gil A. (2019). Effects of Sweeteners on the Gut Microbiota: A Review of Experimental Studies and Clinical Trials. Adv. Nutr..

[29] Food Standards Agency https://www.food.gov.uk/business-guidance/approved-additives-and-e-numbers.

[30] FAO http://www.fao.org/gsfaonline/additives/index.html.

[31] Jagan Mohan Rao L., Ramalakshmi K., Jagan Mohan Rao L., Ramalakshmi K. (2011). Ingredients of soft drinks. Recent Trends in Soft Beverages.

[32] Helstad S., Serna-Saldivar S. (2019). Corn Sweeteners. Corn. Chemistry and Technology.

[33] The American Dietetic Association (2004). Position of the American Dietetic Association: Use of Nutritive and Nonnutritive Sweeteners. J. Am. Diet. Assoc..

[34] Socolovsky S. (2018). Generalidades, seguridad y aprobacion de los edulcorantes no caloricos. Rev. Mex. Neurocienc..

[35] Durán S., Cordón K., Rodríguez M.P. (2013). Edulcorantes no nutritivos, riesgos, apetito y ganancia de peso. Rev. Chil. Nutr..

[36] Rippe J.M., Sievenpiper J.L., Lê K.A., White J.S., Clemens R., Angelopoulos T.J. (2017). What is the appropriate upper limit for added sugars consumption?. Nutr. Rev..

[37] González Á.M., González B.A., González E. (2013). Salud dental: Relación entre la caries dental y el consumo de alimentos. Nutr. Hosp..

[38] Zamora S., Pérez F. (2013). Importancia de la sacarosa en las funciones cognitivas: Conocimiento y comportamiento. Nutr. Hosp..

[39] FAO/OMS http://www.fao.org/gsfaonline/docs/CXS_192s.pdf.

[40] University of Florida https://edis.ifas.ufl.edu/pdffiles/FS/FS18400.pdf.

[41] García F., Gil J., Uribe A., Valencia D. (2019). Dulce veneno, intolerancia hereditaria a la fructosa. Rev. Med..

[42] Bellaera F.A., Hammerschmidt J., Sanz J., Zaccarello D.B., Beccio B. (2018). Jarabe De Maíz De Alta Fructosa, Sus Implicancias En La Salud Y La Información Disponible En El Rotulado De Los Alimentos. Rev. Nutr. Inv..

[43] Parra R.A. (2010). Revisión: Microencapsulación de alimentos. Rev. Fac. Nac. Agron. Medellín..

[44] Souza S.V., Jordão C., Zampieri D., Spontoni B., Leite J., Conceicon A., de Oliveira F., Freitas E. (2020). El consumo de la polidextrosa previene la obesidad y sus comorbilidades en ratas alimentados con dieta hipercalórica. Rev. Chil. Nutr..

[45] Aidoo R.P., Afoakwa E.O., Dewettinck K. (2015). Rheological properties, melting behaviours and physical quality characteristics of sugar-free chocolates processed using inulin/polydextrose bulking mixtures sweetened with stevia and thaumatin extracts. Food Sci. Technol..

[46] Younes M., Aquilina G., Castle L., Engel K.-H., Fowler P., Fürst P., Gürtler R., Gundert-Remy U., Husøy T., Manco M. (2021). Scientific Opinion on the re-evaluation of polydextrose (E 1200) as a food additive. EFSA J..

[47] Bueno-Hernández N., Vázquez-Frías R., Abreu y Abreu A.T., Almeda-Valdés P., Barajas-Nava L.A., Carmona-Sánchez R.I., Chávez-Sáenz J., Consuelo-Sánchez A., Espinosa-Flores A.J., Hernández-Rosiles V. (2019). Revisión de la evidencia científica y opinión técnica sobre el consumo de edulcorantes no calóricos en enfermedades gastrointestinales. Rev. Gastroenterol. Méx..

[48] Cavagnari B.M. (2019). Edulcorantes no calóricos: Características específicas y evaluación de su seguridad. Arch. Argent. Pediatr..

[49] Villarroel P., Gómez C., Vera C., Torres J. (2018). Almidón resistente: Características tecnológicas e intereses fisiológicos. Rev. Chil. Nutr..

[50] FAO http://www.fao.org/tempref/codex/Meetings/CCFA/CCFA47/fa47_03s.pdf.

[51] Geidl-Flueck B., Hochuli M., Németh A., Eberl A., Derron N., Köfeler H.C., Tappy L., Berneis K., Spinas G.A., Gerber P.A. (2021). Fructose- and sucrose- but not glucose-sweetened beverages promote hepatic de novo lipogenesis: A randomized controlled trial. J. Hepatol..

[52] Li X., Yang L., Xu M., Qiao G., Li C., Lin L., Zheng G. (2021). *Smilax china* L. polyphenols alleviates obesity and inflammation by modulating gut microbiota in high fat/high sucrose diet-fed C57BL/6J mice. J. Funct. Foods..

[53] Sun S., Araki Y., Hanzawa F., Umeki M., Kojima T., Nishimura N., Ikeda S., Mochizuki S., Oda H. (2021). High sucrose diet-induced dysbiosis of gut microbiota promotes fatty liver and hyperlipidemia in rats. J. Nutr. Biochem..

[54] Debray F.G., Seyssel K., Fadeur M., Tappy L., Paquot N., Tran C. (2021). Effect of a high fructose diet on metabolic parameters in carriers for hereditary fructose intolerance. Clin. Nutr..

[55] Soltani Z., Rasheed K., Kapusta D.R., Reisin E. (2013). Potential Role of Uric Acid in Metabolic Syndrome, Hypertension, Kidney Injury, and Cardiovascular Diseases: Is It Time for Reappraisal?. Curr. Hypertens. Rep..

[56] Siqueira J.H., Mill J.G., Velasquez-Melendez G., Moreira A.D., Barreto S.M., Benseñor I.M., Molina M.D.C.B. (2018). Sugar-Sweetened Soft Drinks and Fructose Consumption Are Associated with Hyperuricemia: Cross-Sectional Analysis from the Brazilian Longitudinal Study of Adult Health (ELSA-Brasil). Nutrients.

[57] Olaniyi K.S., Olatunji L.A. (2019). Inhibition of pyruvate dehydrogenase kinase-4 by l-glutamine protects pregnant rats against fructose-induced obesity and hepatic lipid accumulation. Biomed. Pharmacother..

[58] Das U.N. (2015). Sucrose, fructose, glucose, and their link to metabolic syndrome and cancer. Nutrition.

[59] Keim N.L., Stanhope K.L., Havel P.J., Caballero B., Finglas P.M., Toldrá F. (2016). Fructose and High-Fructose Corn Syrup. Encyclopedia of Food and Health.

[60] Espinosa J.A., Baxter J., Dash A., Pratt J.W. (2020). Impact of Low Dose Maltodextrin/Citrulline Loading on the Severity and Duration of Stress Induced Hyperglycemia in Cardiac Surgery. J. Am. Coll. Surg..

[61] Hiel S., Gianfrancesco M.A., Rodriguez J., Portheault D., Leyrolle Q., Bindels L.B. (2020). Link between gut microbiota and health outcomes in inulin -treated obese patients: Lessons from the Food4Gut multicenter randomized placebo-controlled trial. Clin. Nutr..

[62] Kending M.D., Lin C.S., Beilharz J.E., Rooney K.B., Boakes R.A. (2014). Maltodextrin can produce similar metabolic and cognitive effects to those of sucrose in the rat. Appetite.

[63] Laudisi F., Di Fusco D., Dinallo V., Stolfi C., Di Grazia A., Marafini I., Colantoni A., Ortenzi A., Alteri C., Guerrieri F. (2019). The Food Additive Maltodextrin Promotes Endoplasmic Reticulum Stress–Driven Mucus Depletion and Exacerbates Intestinal Inflammation. Cell. Mol. Gastroenterol. Hepatol..

[64] Hull S., Re R., Tiihonen K., Viscione L., Wickham M. (2012). Consuming polydextrose in a mid-morning snack increases acute satiety measurements and reduces subsequent energy intake at lunch in healthy human subjects. Appetite.

[65] Ibarra A., Astbury N.M., Olli K., Alhoniemi E., Tiihonen K. (2015). Effects of polydextrose on different levels of energy intake. A systematic review and meta-analysis. Appetite.

[66] Ibarra A., Olli K., Pasman W., Hendriks H., Alhoniemi E., Raza G.S., Herzig K.-H., Tiihonen K. (2017). Effects of polydextrose with breakfast or with a midmorning preload on food intake and other appetite-related parameters in healthy normal-weight and overweight females: An acute, randomized, double-blind, placebo-controlled, and crossover study. Appetite.

[67] Da Silva J.M., Klososki S.J., Silva R., Lorenzo R.S., Silva M.C., Freitas M.Q., Barao C.E., Colombo T. (2020). Passion fruit-flavored ice cream processed with water-soluble extract of rice by-product: What is the impact of the addition of different prebiotic components?. LWT.

[68] Wang H., Ren P., Mang L., Shen N., Chen J., Zhang Y. (2019). In vitro fermentation of novel microwave-synthesized non-digestible oligosaccharides and their impact on the composition and metabolites of human gut microbiota. J. Funct. Foods..

[69] Garavaglia M.B., Rodríguez V., Zapata M.E., Rovirosa A., González V., Flax F., Carmuega E. (2018). Edulcorantes no nutritivos: Consumo de los niños y adolescentes, y alimentos que los aportan. Arch. Argent. Pediatr..

[70] Purohit V., Mishra S. (2018). The truth about artificial sweeteners–Are they good for diabetics?. Indian Heart J..

[71] Bian X., Chi L., Gao B., Tu P., Ru H., Lu K. (2017). The artificial sweetener acesulfame potassium affects the gut microbiome and body weight gain in CD-1 mice. PLoS ONE.

[72] Wang J., Zhao H., Wang Y., Lau H., Zhou W., Chen C., Tan S. (2020). A review of stevia as a potential healthcare product: Up-to-date functional characteristics, administrative standards and engineering techniques. Trends Food. Sci. Technol..

[73] Yalamanchi S., Srimath R., Dobs A., Caballero B., Finglas P.M., Toldrá F. (2016). Acesulfame-K. Encyclopedia of Food and Health.

[74] Magnuson B.A., Roberts A., Nestmann E.R. (2017). Critical review of the current literature on the safety of sucralose. Food Chem. Toxicol..

[75] Myint K.Z., Wu K., Xia Y., Fan Y., Shen J., Zhang P., Gu J. (2020). Polyphenols from Stevia rebaudiana (Bertoni) leaves and their functional properties. J. Food Sci..

[76] Singh D.P., Kumari M., Prakash H.G., Rao G.P., Solomon S. (2019). Phytochemical and Pharmacological Importance of Stevia: A Calorie-Free Natural Sweetener. Sugar Tech..

[77] Silva M.R., Moya C.Á., León A.G.S., Velasco R.R., Flores A.M.C. (2018). Genotoxic activity of saccharin, acesulfame-K, stevia and aspartame-acesulfame-K in commercial form. J. Clin. Toxicol..

[78] Chappell G.A., Wikoff D.S., Doepker C.L., Borghoff S.J. (2020). Lack of potential carcinogenicity for acesulfame potassium–Systematic evaluation and integration of mechanistic data into the totality of the evidence. Food Chem. Toxicol..

[79] Kumar N., Singh A., Sharma D.K., Kishore K., Singh R.L., Mondal S. (2019). Toxicity of Food Additives. Food Safety and Human Health.

[80] Li W., Liu Y., Wang Y., Li X., Liu X., Guo M., Tan Y., Qin X., Wang X., Jiang M. (2020). Sucralose Promotes Colitis-Associated Colorectal Cancer Risk in a Murine Model Along With Changes in Microbiota. Front. Oncol..

[81] Paredes A.E., Naranjo M.C. (2016). La Stevia rebaudiana como coadyuvante en la prevención y el control de la caries dental: Una revisión de literatura. Acta Odontol. Colomb..

[82] Ruyter J., Olthof M., Seidell J., Katan B. (2012). A trial of sugar-free or sugar-sweetened beverages and body weight in children. N. Engl. J. Med..

[83] Farid A., Hesham M., El-Dewak M., Amin A. (2020). The hidden hazardous effects of stevia and sucralose consumption in male and female albino mice in comparison to sucrose. Saudi Pharm. J..

[84] DeMartino P., Cockburn D.W. (2020). Resistant starch: Impact on the gut microbiome and health. Curr. Opin. Biotechnol..

[85] Togo J., Hu S., Li M., Niu C., Speakman J.R. (2019). Impact of dietary sucrose on adiposity and glucose homeostasis in C57BL/6J mice depends on mode of ingestion: Liquid or solid. Mol. Metab..

[86] Karjalainen S., Tolvanen M., Pienihäkkinen K., Söderling E., Lagström H., Simell O., Niinikoski H. (2015). High sucrose intake at 3 years of age is associated with increased salivary counts of mutans streptococci and lactobacilli, and with increased caries rate from 3 to 16 years of age. Caries Res..

[87] Van Opstal A.M., Kaal I., van den Berg-Huysmans A.A., Hoeksma M., Blonk C., Pijl H., Rombouts S., van der Grond J. (2019). Dietary sugars and non-caloric sweeteners elicit different homeostatic and hedonic responses in the brain. Nutrition.

[88] Higgins K.A., Mattes R.D. (2019). A randomized controlled trial contrasting the effects of 4 low-calorie sweeteners and sucrose on body weight in adults with overweight or obesity. Am. J. Clin. Nutr..

[89] Maersk M., Belza A., Stødkilde-Jørgensen H., Ringgaard S., Chabanova E., Thomsen H., Pedersen S.B., Astrup A., Richelsen B. (2012). Sucrose-sweetened beverages increase fat storage in the liver, muscle, and visceral fat depot: A 6-mo randomized intervention study. Am. J. Clin. Nutr..

[90] Aijälä M., Malo E., Ukkola O., Bloigu R., Lehenkari P., Autio-Harmainen H., Santaniemi M., Kesäniemi Y.A. (2013). Long-term fructose feeding changes the expression of leptin receptors and autophagy genes in the adipose tissue and liver of male rats: A possible link to elevated triglycerides. Genes Nutr..

[91] Johnson R.J., Perez-Pozo S.E., Lillo J.L., Grases F., Schold J.D., Kuwabara M., Sato Y., Hernando A.A., Garcia G., Jensen T. (2018). Fructose increases risk for kidney stones: Potential role in metabolic syndrome and heat stress. BMC Nephrol..

[92] Olguin M.C., Posadas M.D., Revelan G.C., Labourdette V., Marinozzi D.O., Venezia M.R., Zingale M.I. (2015). Efectos del consumo elevado de fructosa y sacarosa sobre parámetros metabólicos en ratas obesas y diabéticas. Rev. Chil. Nutr..

[93] Stanhope K.L., Medici V., Bremer A.A., Lee V., Lam H.D., Nunez M.V., Chen G.X., Keim N.L., Havel P.J. (2015). A dose-response study of consuming high-fructose corn syrup-sweetened beverages on lipid/lipoprotein risk factors for cardiovascular disease in young adults. Am. J. Clin. Nutr..

[94] Meyers A.M., Mourra D., Beeler J.A. (2017). High fructose corn syrup induces metabolic dysregulation and altered dopamine signaling in the absence of obesity. PLoS ONE.

[95] Thomson P., Santibañez R., Aguirre C., Galgani J.E., Garrido D. (2019). Short-term impact of sucralose consumption on the metabolic response and gut microbiome of healthy adults. Br. J. Nutr..

[96] Anton S.D., Martin C.K., Han H., Coulon S., Cefalu W.T., Geiselman P., Williamson D.A. (2010). Effects of stevia, aspartame, and sucrose on food intake, satiety, and postprandial glucose and insulin levels. Appetite.

[97] Farhat G., Berset V., Moore L. (2019). Effects of Stevia Extract on Postprandial Glucose Response, Satiety and Energy Intake: A Three-Arm Crossover Trial. Nutrients.

[98] Bonnet F., Tavenard A., Esvan M., Laviolle B., Viltard M., Lepicard E.M., Lainé F. (2018). Consumption of a Carbonated Beverage with High-Intensity Sweeteners Has No Effect on Insulin Sensitivity and Secretion in Nondiabetic Adults. J. Nutr..

[99] Cameron I., Alam M.A., Wang J., Brown L. (2012). Endurance exercise in a rat model of metabolic syndrome. Can. J. Physiol. Pharmacol..

[100] Barquera S., Rivera J.A. (2020). Obesity in Mexico: Rapid epidemiological transition and food industry interference in health policies. Lancet Diabetes Endocrinol..

[101] Jimenez P., Ochoa A. (2015). Obesidad: ¿Cómo afecta nuestra salud lo que mamá comió durante el embarazo?. Revista milenaria. Cienc. yarte.

[102] Vázquez M.C., Guevara R.G., Aguirre H., Alvarado A.M., Romero H. (2017). Current consumption of natural sweeteners (benefits and problems): Stevia. Rev. Med. Electrón..

[103] Aldrete-Velasco J.A., Aranceta-Bartrina J., Rodríguez-García J.A., Durán-Coyote S., Pedraza-Chavéz J., Reyes-Zavala C. (2020). Conocimiento, consumo y recomendación de edulcorantes no calóricos en una población de profesionales de la salud en México. Med. Int. Mex..

[104] Romo A., Almeda P., Brito G.X., Gómez F.J. (2017). Prevalencia del consumo de edulcorantes no nutritivos (ENN) en una población de pacientes con diabetes en México. Gac. Med. Mex..

[105] Tolentino L., Rincón S., Bahena L., Ríos V., Barquera S. (2018). Conocimiento y uso del etiquetado nutrimental de alimentos y bebidas industrializados en México. Salud. Publ. Mex..

[106] Durán S., Quijada M., Silva L., Almonacid N., Berlanga M., Rodríguez M. (2011). Niveles de ingesta diaria de edulcorantes no nutritivos en escolares de la región de valparaíso. Rev. Chil. Nutr..

[107] Muñoz I., Sevilla M.D., García F.E., Sanchéz J.G., Sánchez L.G. (2020). Bebidas edulcorantes y su riesgo para la salud. Rev. Educ. Cienc. Ing..

[108] Cairns G., Angus K., Hastings G., Caraher M. (2013). Systematic reviews of the evidence on the nature, extent and effects of food marketing to children. A retrospective summary. Appetite.

[109] Tatlow-Golden M., Garde A. (2020). Digital food marketing to children: Exploitation, surveillance and rights violations. Glob. Food Secur..

